# Non-Coding RNAs in the Therapeutic Landscape of Pathological Cardiac Hypertrophy

**DOI:** 10.3390/cells11111805

**Published:** 2022-05-31

**Authors:** Joana Silva, Paula A. da Costa Martins

**Affiliations:** 1 Department of Cardiology, CARIM School for Cardiovascular Diseases, Faculty of Health, Medicine and Life Sciences, Maastricht University, 6229 ER Maastricht, The Netherlands; j.alves@mirabilis-therapeutics.com; 2 Mirabilis Therapeutics BV, 6229 EV Maastricht, The Netherlands; 3 Department of Physiology and Cardiothoracic Surgery, Faculty of Medicine, University of Porto, 4200-319 Porto, Portugal

**Keywords:** non-coding RNAs, cardiac hypertrophy, cardiac pathological remodeling, antisense oligonucleotide-based therapeutics, drug delivery

## Abstract

Cardiovascular diseases are a major health problem, and long-term survival for people diagnosed with heart failure is, still, unrealistic. Pathological cardiac hypertrophy largely contributes to morbidity and mortality, as effective therapeutic approaches are lacking. Non-coding RNAs (ncRNAs) arise as active regulators of the signaling pathways and mechanisms that govern this pathology, and their therapeutic potential has received great attention in the last decades. Preclinical studies in large animal models have been successful in ameliorating cardiac hypertrophy, and an antisense drug for the treatment of heart failure has, already, entered clinical trials. In this review, we provide an overview of the molecular mechanisms underlying cardiac hypertrophy, the involvement of ncRNAs, and the current therapeutic landscape of oligonucleotides targeting these regulators. Strategies to improve the delivery of such therapeutics and overcome the actual challenges are, also, defined and discussed. With the fast advance in the improvement of oligonucleotide drug delivery, the inclusion of ncRNAs-targeting therapies for cardiac hypertrophy seems, increasingly, a closer reality.

## 1. Introduction

In a constantly changing world, new and intimidating diseases continue to emerge, and, yet, none overcomes the burden of cardiovascular diseases (CVDs), which are often worsening the prognosis of some others. CVDs are the major cause of death worldwide, according to the World Health Organization, with heart failure (HF) actively contributing to high mortality rates. Among the different cardiac conditions culminating in HF, cardiac hypertrophy is a very common one [1,2].

Cardiac hypertrophy is the heart’s response to increased hemodynamic load. It is characterized by an increase in myocardium mass, as a compensatory mechanism of the heart, to minimize wall stress and maintain contractile function [3,4,5,6,7]. A prolonged or persistent increase in hemodynamic demand, often, causes decompensated or pathological hypertrophy. Such chronic structural remodeling of the left ventricle (LV)—left ventricular hypertrophy (LVH)—is among the most robust driving forces for cardiovascular morbidity and mortality, being strongly associated with the development of chronic heart failure [8,9,10].

LVH can be concentric or eccentric, according to the geometrical changes occurring in this chamber. An eccentric hypertrophy is, usually, a consequence of volume overload, often associated with valvular regurgitation. Depending on the severity and duration of such overload, this can result in a progressive LV enlargement and consequent reduced LV systolic function. On the other hand, LV pressure overload can culminate in concentric hypertrophic growth, where both the LV mass and the LV wall thickness are enlarged. This pathological scenario, usually, involves reduced LV diastolic function and diastolic HF [11,12].

**Figure 1:**
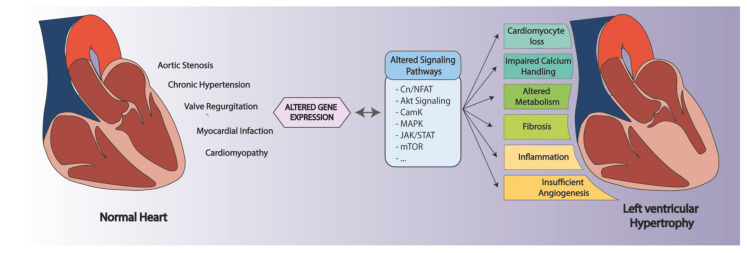
Schematic representation of the mechanisms involved in the development of pathological left ventricular hypertrophy.

While the duration of the cardiac stress is not the deciding factor between an adaptive or a pathological response, the nature of the stimuli and the affected signaling pathways are the determinants of the outcome [1,13], with cardiac pressure overload being able to induce pathological hypertrophy, even when intermittent [14]. The major causes leading to disease include aortic stenosis and chronic hypertension [15,16], associated with a series of molecular and pathophysiological changes, including cardiomyocyte loss, calcium handling, metabolism, sarcomere organization, fibrosis, inflammation, and angiogenesis. These are, usually, a result of alterations in signaling pathways and, consequently, in gene expression [1,13,17]. A schematic representation of the described mechanisms can be found in Figure 1.

Despite its close association with HF, effective therapies targeting cardiac hypertrophy are lacking. New therapeutic strategies arise on the horizon, aiming to specifically target the intrinsic mechanisms involved in pathological remodeling. In the current era, there has been a shift towards molecular-based therapies, as these have shown improved clinical results, overcoming the effectiveness of those targeting observable symptoms only. Non-coding RNAs, with their versatility to regulate different signaling pathways, are promising therapeutic targets for such complex pathologies and have been confirming their place in this field in the last decade. Here, below, we review the most relevant regulatory non-coding RNAs and their therapeutic potential in the different mechanisms involved in concentric cardiac hypertrophy.

## 2. Non-Coding RNAs (ncRNAs)

The concept of RNA, as just the bridge between the coding information on DNA and protein synthesis, is no longer up to date. Recent studies suggest that about two-thirds of the mammalian genome is actively transcribed, with only 1.9% encoding for proteins and about 98% of the transcribed RNA having no coding potential [18,19]. These ncRNAs play key roles in DNA replication, transcriptional and post-transcriptional gene expression, chromatin processing, and maintaining the stability of mRNA and genomic integrity [19,20]. Certain ncRNAs are considered housekeeping RNAs, endogenously expressed to ensure normal cell functioning, and include small nuclear RNAs (snRNAs), small nucleolar RNAs (snoRNAs), transfer RNAs (tRNAs), ribosomal RNAs (rRNAs), and guide RNAs (gRNAs) [18]. However, of singular importance for current clinical research, is the fraction of ncRNAs with strong regulatory abilities in pathophysiological processes.

Regulatory ncRNAs include short-interfering RNAs (siRNAs), tRNA-derived fragments (tRFs), piwi-interacting RNAs (piRNAs), microRNAs (miRNAs or miRs), and long non-coding RNAs (lncRNAs), which, also, include circular RNAs (circRNAs) [21,22]. During the last decade, associations between ncRNAs and (epigenetic) regulation of several diseases are increasing. Regarding cardiac hypertrophy, miRNAs, lncRNAs, and circRNAs are, currently, the most studied ncRNAs, regarding their therapeutic potential [21].

MicroRNAs are endogenous small noncoding RNAs, single-stranded, of 21–23 nucleotides in length. These are encoded by genes in various locations throughout the genome, either independent or organized in clustered transcriptional units [23], and are considered vital evolutionary components of gene regulation, as they are highly conserved between mammals [24]. miRNAs can post-transcriptionally regulate gene expression, by binding their seed region (2–8 nucleotides) to the complementary 3′-untranslated regions (3′-UTR) of the target messenger RNA (mRNAs). When the complementarity is total, the mRNA is cleaved, but, if only partially, the translation of the target mRNA is restrained, with the stability of the mRNA being uncompromised. This non-mandatory complete complementarity enables miRNAs to target different mRNAs and modulate different gene-expression programs and pathways, simultaneously. Likewise, one single messenger RNA can be regulated by multiple miRNAs at the same time [21].

MicroRNAs have shown not only a therapeutic but also a diagnostic potential. Circulating microRNAs are gaining increasing interest, since they were found to be remarkably stable, to regulate gene expression in distant targets by mediating cell-to-cell communication and, mostly, due to their potential as diagnostic biomarkers [25]. Distinctive patterns of circulating miRNAs were found in several cardiovascular diseases, including pathological cardiac hypertrophy [26].

On the other hand, lncNAs are a very heterogeneous group of regulatory ncRNAs, with more than 200 nucleotides in length. Their heterogeneity is a consequence of differences in their location, sequence structure, inherent characteristics, and mechanisms of action [27]. lncRNAs are the most abundant ncRNAs in the mammalian transcriptome, and their mechanistic characterization is, still, in its beginning, with only a small number of lncRNAs having been characterized in-depth and, functionally, verified in vivo [28]. In contrast to miRNAs, lncRNA transcripts seem to be less conserved among species [29]. lncRNAs are associated with a diverse range of functions, including structural functions, regulation of gene expression, both in *cis* and in *trans*, post-transcriptional regulation, and regulation of chromatin modifications [19]. Thus, it is not surprising that changes in lncRNA expression or activity have been associated with several diseases because of their extensive regulatory control. lncRNAs have, also, been described as crucial regulators of cardiomyocyte maturation, highlighting their potential in regenerative medicine [30].

Circular RNAs (circRNAs), as the name indicates, display a circular structure resulting from a covalent linkage between the 3′ and 5′ ends, which provides them increased stability, as the lack of exposed ends makes them less prone to degradation [31]. circRNAs are highly evolutionary conserved, and their expression can be associated with certain cell-types and/or developmental stages [21,31]. Although sometimes regarded as lncRNAs with sizes ranging from 100 to over 4000 nucleotides [31], due to their special characteristics, cirRNAs deserve their own identity. They, normally, result from splicing events and circularization of introns or exons and are found in the cytoplasm, where they can have different functions, including acting as sponges for miRNAs, regulating gene expression mediated by RNA binding proteins (RBPs), and regulating cleavage or gene transcription processes [21].

Although piRNAs were thought to be mainly functional at the germline, they were recently reported as abundantly expressed in cardiomyocytes [32]. PiRNAs are a class of small ncRNAs that despite remaining poorly described, have, already, been recognized as regulators of cardiac pathophysiology. They are longer than microRNAs, with a length between 24 and 31 nucleotides, and are, evolutionarily, less conserved. piRNAs act as the guides of a class of Argonaute (AGO) proteins, the PIWI proteins, which are involved in the maintenance of genomic integrity and stability, as well as in the regulation of the epigenetic state and germ cell function [33]. Besides their role as potential biomarkers, piRNAs have, also, been suggested as potential therapeutic targets. piRNAs, whose expression was altered in pressure overloaded mice hearts were identified, including upregulation of piR-6999 and piR-110550 as well as downregulation of piR-106654 and piR-13375. However, cardiac-hypertrophy-associated piRNA (CHAPIR), described later in this paper, was the only piRNA showing an evident effect on cardiomyocyte hypertrophy, so far [34].

Finally, tRFs are an emerging class of small ncRNAs, produced from the cleavage of mature tRNA, by stress-released ribonucleases. These fragments have been described to act as siRNAs, being able to modulate several biological processes [35]. The involvement of tRFs in cardiac hypertrophy was reported, once they were found markedly enriched in a rat model of isoproterenol-induced cardiac hypertrophy, with overexpression of tRFs1 and tRFs2 leading to increased cardiomyocyte size and expression of hypertrophic markers in vitro. Furthermore, tRFs 1, 2, 3, and 4 were upregulated in both hypertrophic parental and offspring hearts, with the hypertrophic offspring showing increased expression of *MYH7* and *nppa* genes as well as increased cardiac fibrosis and apoptosis. These results suggest that tRFs play an important role in cardiac hypertrophy, and their epigenetic regulation contributes to the inheritance of cardiac hypertrophy across generations [36].

There are already several in vitro and in vivo studies, showing the role of such regulatory ncRNAs in pressure-overload-induced cardiac hypertrophy. For the purpose of this paper, we focus on the in vivo findings, since these provide a better translational perspective of their therapeutic relevance.

## 3. ncRNAs in Pressure-Overload-Induced Cardiac Hypertrophy

In pressure-overload-induced cardiac hypertrophy, specific alterations in gene expression are taking place. Expression of fetal genes, including the ones encoding natriuretic peptide A (ANP), natriuretic peptide B (BNP), myosin heavy chain cardiac muscle *β*-isoform (MYHCβ or MYH7) and skeletal muscle α-actin (ACTA1), is a characteristic of a morbid remodeling [13,17]. Moreover, mutations in genes encoding for sarcomeric proteins were, also, reported in hypertrophic cardiomyopathy, leading to hypertrophy and cardiac dysfunction [37]. On one hand, altered gene expression is normally a consequence of dysregulation of signaling pathways, usually culminating in the amplified expression of pro-hypertrophic genes. On the other hand, these pro-hypertrophic genes will again, affect the same and/or other signaling pathways, creating a loop between altered signaling and altered gene expression.

Several ncRNAs have been reported to regulate the expression of pro- and anti-hypertrophic gene programs. miR-92b-3p was downregulated, both in a rat model of Angiotensin II (AngII)-induced hypertrophy and in the myocardium of patients with cardiac hypertrophy. This miRNA was found to directly repress myocyte enhancer factor 2 (*MEF2D*) [38]. *MEF2D* is a known mediator of cardiac hypertrophy, with its overexpression being enough to drive pathological cardiac remodeling in mice [39]. Recent findings showed that the miR-106~25 cluster, to which miR-92b-3p belongs, targets the pro-hypertrophic transcription factors MEF2D and heart and neural crest derivatives-expressed 2 (Hand2). This cluster is downregulated in end-stage HF human samples, and its silencing exacerbates hypertrophic growth in mice subjected to transverse aortic constriction (TAC). Interestingly, in vivo overexpression of the miR-106~25 cluster, also, resulted in cardiac enlargement, but with no signs of pathological remodeling or dysfunction. In contrast, genetic deletion of this cluster resulted in spontaneous and exaggerated hypertrophic remodeling in mice, by upregulation of MEF2D and Hand2. Thus, this cluster seems to regulate a sensitive balance of cardiomyocyte growth between hyperplasia and hypertrophy [40].

Recent work showed that miR-145-5p worsens pathological response in AngII-treated cardiomyocytes, by decreasing the expression of activating transcription factor 3 (ATF3). ATF3 is part of the cAMP response element-binding protein/ATF family, and its expression has shown protective effects in hypertensive hearts [41,42]. In this study, inhibition of this miRNA showed restoration of ATF3 levels and consequent attenuation of pressure overload-induced hypertrophy [41]. Interestingly, circ_nuclear factor I X (circNfix), reportedly, sponged miR-145-5p and abolished its detrimental effect on inhibiting ATF3 expression. Overexpression of this circRNA reversed cardiac hypertrophy in mice subjected to TAC [41].

lncRNAs, such as myosin heavy-chain-associated RNA transcript (Mhrt) and cardiac-hypertrophy-associated epigenetic regulator (Chaer), are, also, regulating the expression of gene programs in concentric cardiac hypertrophy. The cardiac-specific lncRNA Mhrt is the antisense transcript of *MYH7*. A cardioprotective role was reported for this lncRNA in a mice model of hypertrophy: its suppression stimulated the progression of hypertrophy to heart failure, while its pre-overexpression using a transgenic mice line was beneficial, delaying this progression. Mhrt inhibits the action of cardiac stress-activated chromatinremodeling factor (Brg1), which can bind to chromatinized DNA but not naked DNA. Mhrt, competitively, binds to helicase and prevents chromatin remodeling, so Brg1 can no longer act on its target hypertrophic genes, decreasing cardiac hypertrophy [43]. The lncRNA Chaer, on the other hand, directly interacts with and downregulates enhancer of zeste homolog 2 (*EZH2*), an anti-hypertrophic gene. This results in negative regulation of polycomb repressor complex 2 (PRC2). PRC2 is normally responsible for the di- or trimethylation of H3K27, at the promoter regions of pro-hypertrophic genes. Thus, by negatively regulating this modification, Chaer upregulates the expression of pro-hypertrophic genes. This lncRNA was found necessary for the development of cardiac hypertrophy, and its transcript levels were significantly increased in myocardial samples, from patients with dilated cardiomyopathy. Although Chaer-knockout mice subjected to TAC presented a significantly attenuated hypertrophic response, its knockdown by siRNA injections in mice was effective in suppressing hypertrophy, when administered before TAC, but not when applied after, suggesting the existence of a critical window of epigenetic regulation [44].

Novel candidate genes are being reported to be involved in cardiac disease, which is the case of the carboxypeptidase X member 2 (*CPXM2*) gene that was, recently, associated with hypertension-induced hypertrophy. Although the role of *CPXM2* is still, widely, unexplored, its expression seems to be directly regulated by miR-29b and miR-497 in rat cardiomyocytes, corroborating the vast potential of ncRNAs in cardiac research [45].

Table 1 presents an overview of the ncRNAs involved in the regulation of gene programs involved in pressure overload-induced cardiac hypertrophy.

ncRNAs can interfere with gene expression programs, through different mechanisms and signaling pathways, and, although these are often not well understood [46], their regulatory role in cardiac hypertrophy has been reported.

### 3.1. ncRNAs Involved in Specific Cardiac Hypertrophy-Related Pathways

Once regulatory ncRNAs can interfere with different signaling pathways, simultaneously, the same ncRNA is often associated with more than one molecular mechanism. Signaling pathways implicated in cardiac hypertrophy include Ca^2+^/Calcineurin (Cn)/nuclear factor of activated T cells (NFAT) and Ca^2+^/Calmodulin-dependent kinase II (CaMKII), the mitogen-activated protein kinase (MAPK), comprising extracellular signal-regulated kinases 1/2 and 5 (ERK1/2 and 5), p38 MAPK, c-Jun NH2-terminal kinase (JNK), and the janus kinase/signal transducers and activators of transcription (JAK/STAT) [47,48,49]. Wnt, AngII, protein kinase B (Akt), tumor necrosis factor-α (TNF-α), and β-arrestin-2 have, also, been suggested to have a crucial role in the process [48].

#### 3.1.1. Calcineurin/NFAT Signaling

The calcineurin/NFAT signaling pathway has been, widely, associated with concentric cardiac hypertrophy, and several ncRNAs play a role in its regulation. miR-1, one of the best-described miRNAs regarding cardiac remodeling, can regulate several pathways involved in disease progression. Ikeda et al. showed that miR-1 modulates cardiomyocyte growth and reduces cardiomyocyte hypertrophy, by downregulating the expression of calmodulin (CaM), Mef2a, and GATA Binding Protein 4 (GATA4) and attenuating calcineurin/NFAT signaling [50].

miR-1 and miR-133 are, often, studied in combination, since they are clustered on the same chromosomal loci andare specifically expressed in skeletal muscle and cardiomyocytes [51], plus their levels are reduced in mouse and human samples of cardiac hypertrophy. AntagomiR-mediated silencing of miR-133 in mice led to an aggravated hypertrophic response, while its overexpression by adenoviral infection resulted in ameliorated cardiac function [52]. miR-133 was later found to be involved in post-transcriptional repression of Cn/NFAT signaling. Inhibition of calcineurin in diseased mice resulted in increased levels of this microRNA, which was accompanied by an improved phenotype [53]. In line with these studies, circSLC8A1-1, a circRNA described to play a pro-hypertrophic role by acting as a sponge of miR-133, induces heart failure when overexpressed and attenuates cardiac hypertrophy and progression towards failure, when inhibited in mice [54]. A more recent study, by Qian et al. (2021), showed that METTL3-mediated N6- methyladenosine (m6A) modification is, also, promoting the repression of miR-133a during cardiac hypertrophy, suggesting a new therapeutic target to restore its protective mechanism [55].

The recently identified piRNA CHAPIR, or piR-141981, was shown to target the m6A methylation of Poly(ADP-Ribose) polymerase family member 10 (*Parp10*) mRNA transcripts. By blocking its methylation, CHAPIR increases the expression of PARP10 which, in turn, inhibits the activity of glycogen synthase kinase 3β (GSK3β), leading to the accumulation of NFATC4 in the nucleus and the development of pathological hypertrophy. Administration of a CHAPIR mimic aggravated the hypertrophic response in mice subjected to pressure overload, while CHAPIR deletion significantly attenuated hypertrophy, with consequent improvement of heart function [34].

miR-199b was reported as sufficient to activate the pro-hypertrophic calcineurin/NFAT signaling pathway, thus inducing a severe hypertrophic and pathological remodeling in two different murine models of pressure overload. Treatment with a specific antagomiR resulted in improved hypertrophic response and cardiac function [56].

Finally, Forkhead box transcription factors, such as the O subfamily (FoxO), have been described as repressors of cardiac hypertrophy, through inhibition of the calcineurin/NFAT signaling cascade. Moreover, FoxO1 and FoxO3 were found to be phosphorylated and, hence, inactivated in pressure-overloaded hearts [57]. Inactivation of FoxO3 can be mediated by miR-182 [58,59] and miR-23a [60], and in vivo inhibition of both microRNAs resulted in attenuated hypertrophic response in diseased mice. miR-212/132 can, also, regulate the FoxO3/calcineurin/NFAT pathway, with their overexpression inducing pathological cardiac hypertrophy in mice, by hyper activating calcineurin/NFAT signaling [61]. Inhibition of miR-132 in a large animal model of cardiac hypertrophy resulted in milder hypertrophic and fibrotic responses as well as improved cardiac function [62]. A recent study has, however, shown that miR-212 modulates left ventricle hypertrophy, through different mechanisms in chronic kidney disease, as calcineurin/NFAT was not affected in nephrectomized rats overexpressing miR-212 [63]. miR-30 expression was, recently, shown to attenuate LV hypertrophy in chronic kidney-diseased rats [64]. Unique features are foreseen, for this form of LV hypertrophy.

#### 3.1.2. CaMKII Signaling

CaMKII signaling pathways have, also, been implicated in maladaptive cardiac remodeling. They can be the target of ncRNAs such as miR-675, lncRNA H19, lncRNA TINCR, and miR-214.

miR-675 directly targets and downregulates calmodulin-dependent protein kinase II delta (CaMKIIδ). Inhibition of this microRNA triggered exacerbated hypertrophic phenotypes in a mouse model of TAC, through upregulation of CaMKII signaling [65]. lncRNA H19 encodes miR-675, since exon 1 of H19 has a miRNA-containing hairpin that acts as template for miR-675. Thus, CaMKIIδ is not only regulated by miR-675 but also by the H19-miR-675 axis. The levels of H19 were increased, both in mice subjected to TAC and human samples of failing hearts. Overexpression of H19 was able to reduce growth and expression of hypertrophic genes in mouse neonatal cardiomyocytes, subjected to a hypertrophic stimulus. On the other hand, inhibition of H19-miR-675 axis exacerbated cardiac hypertrophy in vivo [65].

Upregulation of the lncRNA Terminal Differentiation Inducing Non-coding RNA (TINCR) was shown to reduce cardiac hypertrophy when upregulated, while lower expression levels are associated with worsened phenotypes. TINCR directly combines and promotes the activity of the anti-hypertrophic gene *EZH2*, which can epigenetically silence CaMKII through the tri-methylation of histone H3 lysine 27 methylation (H3K27me3). Thus, increased levels of TINCR reduced CaMKII levels and, consequently, attenuated cardiomyocyte hypertrophy in mice subjected to TAC [66]. Contrarily, miR-214 showed pro-hypertrophic effects in a pressure-overload mouse model of heart failure, by directly targeting and inhibiting *EZH2* [67].

CaMKII, as the name indicates, will be closely related to calcium-handling processes during cardiac hypertrophy, meaning that the mentioned ncRNAs can be playing a role in related pathophysiological mechanisms.

#### 3.1.3. MAPK Signaling

Different microRNAs regulate cardiac hypertrophy, by targeting MAPK signaling pathways. miR-378 targets MAPK1, kinase suppressor of ras 1 (KSR1), growth factor receptor-bound protein 2 (GRB2), and insulin-like growth factor receptor 1 (IGF1R), and, thus, suppresses MAPK pathways in mouse models of cardiac-pressure overload [68,69]. Restoration of miR-378 expression using adeno-associated virus 9 (AAV9)-mediated gene transfer significantly attenuated hypertrophy and improved cardiac function in pressure-overloaded mouse hearts [69]. In contrast, miR-499 activates this pathway by upregulation of several elements of the MAPK signaling cascade, such as Map2k3, Map3k5, Map3k6, Map4k2, Map4k4, Mapk14, and Mknk2. Moreover, miR-499 alters the phosphorylation of both heat-shock protein 90 (HSP90) and protein phosphatase 1α (Ppp1ca), both known to influence calcium transport, cell survival, and Akt signaling. Overexpression of miR-499 showed detrimental effects in mice subjected to TAC [70].

Other microRNAs targeting MAPK signaling include miR-1 and miR-21. Since their regulatory roles in this pathway affect concretely the fibrotic response, they will be described on Section 3.2.4, where such pathophysiological response is discussed.

#### 3.1.4. Akt Signaling

ncRNAs, often, exert their roles in Akt signaling through regulation of phosphatase and tensin homolog (PTEN), a secondary messenger that prevents the activation of Akt. miR-20b is known to downregulate PTEN, favoring the activation of Akt signaling and the progression of cardiac hypertrophy. On the other hand, cardiac-hypertrophy-associated regulator (CHAR) is a lncRNA that acts as a competitive endogenous RNA (ceRNA) to downregulate miR-20b, upregulate PTEN, and, consequently, decrease Akt signaling. Silencing of CHAR in vivo exacerbates cardiac hypertrophy in mice subjected to TAC [71]. Similarly, also miR-21 [72] and miR-217 [73] are reported to target the PTEN/Akt pathway, by repressing PTEN and increasing Akt activity and, thus, aggravating pressure-overload-induced cardiac hypertrophy.

#### 3.1.5. JAK/STAT and Wnt Signaling Pathways

Other signaling pathways reported to be involved in pathological cardiac hypertrophy include JAK/STAT and Wnt.

JAK/STAT signaling is targeted by miR-148. miR-148 protects cardiomyocytes from hypertrophic growth by downregulating cytokine co-receptor glycoprotein 130 (gp30) and, thus, decreasing the activity of STAT3 hypertrophic signaling. The therapeutic potential of this microRNA was well described in this study, as its silencing by a specific antagomiR led to cardiac dilation, while AAV9-mediated overexpression of miR-148 was able to suppress such phenotypes in mice subjected to TAC. Moreover, overexpression of miR-148, four weeks after TAC, resulted in attenuated transition of concentric to eccentric remodeling [74].

Finally, Wnt signaling is targeted by miR-29. miR-29 plays a role in cardiac hypertrophy, as its silencing can prevent the induction of pathological remodeling in a mouse model of LV pressure overload [75]. This microRNA directly represses GSK3β activity, an essential negative regulator of hypertrophy, which is, also, negatively regulated by Wnt and Cn/NFAT signaling [76]. Interestingly, regulation of miR-29b expression during pressure overload was reported to be opposite in male and female mice: this microRNA was upregulated in TAC females, while downregulated in TAC males. Differential expression of GSK3β culminated in opposite outcomes, with the results suggesting that miR-29b can have a detrimental role in female mice but a cardioprotective role in male mice during LV hypertrophy [77]. Understanding the sex-specific modulation of ncRNAs can, thus, determine the therapeutic outcome.

The activation of the described pathways is associated with mechanical stress and determines the gene expression pattern, which will influence the impairment of several pathophysiological processes and the consequent cardiac deleterious phenotype.

Table 2 summarizes the ncRNAs involved in the different signaling pathways involved in cardiac hypertrophy.

### 3.2. ncRNAs Involved in Specific Pathophysiological Processes

Different processes involved in cardiac hypertrophy include cardiomyocyte remodeling and loss, impaired calcium handling, impaired metabolism, cardiac fibrosis, inflammation, and impaired angiogenesis. Here, we review the ncRNAs that, besides interfering with the known signaling pathways, have, also, been described to play a specific role in such pathophysiological mechanisms.

#### 3.2.1. ncRNAs in Cardiomyocyte Remodeling and Loss

Cardiomyocyte loss is, often, associated with the activation of apoptotic pathways. Apoptosis seems to be targeted by miR-183-5p, which is able to decrease hypertrophic growth in a mouse model of pressure overload, by regulating the expression of C/EBP homologous protein (CHOP), the key inducer of apoptosis [78]. Moreover, the heart-related circRNA (HRCR) was found to play a protective role by acting as a sponge of miR-223, which, when overexpressed, spontaneously induced cardiac hypertrophy and heart failure in mice. This miRNA regulates anti-apoptotic protein apoptosis repressor with caspase recruitment domain (ARC), promoting cardiomyocyte apoptosis. Thus, HRCR exerts its anti-hypertrophic function by restoring APC levels and preventing apoptosis, ameliorating cardiac morphology and function [79].

As an addition to apoptosis, under hypertrophic conditions, cardiomyocytes, usually, benefit from an increase in autophagy, a process where aged proteins and impaired organelles are degraded and recycled, preventing the accumulation of ubiquitinated protein aggregates that, otherwise, would become toxic to the cell [80]. This process, by contributing to the synthesis of new proteins and increase in cardiomyocyte size and sarcomeric remodeling, is considered a protective mechanism against pathological remodeling and cardiomyocyte death [81]. NFAT signaling activates the lncRNA cardiac-hypertrophy-associated transcript (Chast) in pressure-overloaded murine hearts and in hearts of patients with aortic stenosis, which, in turn, regulates the expression of its adjacent protein-coding gene Plekhm1. Plekhm1 is involved in autophagy regulation, and its downregulation results in diminished cardiac autophagy, promoting cardiac hypertrophy. Chast is a promising therapeutic target, as its silencing leads to ameliorated cardiac hypertrophy and improved cardiac function in mice [82].

Despite the evidence of its benefits, autophagy can also be detrimental, since its contribution to cardiomyocyte growth, when dysregulated, can lead to cellular dysfunction and death [83]. Therefore, while some studies suggest beneficial effects in therapies aiming to promote it, others show that it should be prevented. lncRNA Gm15834, for instance, promotes myocardial hypertrophy, by acting as a sponge of the autophagy-suppressor miR-30b-3p. lncRNA Gm15834 was found upregulated in TAC mice hearts, and its upregulation was associated with enhanced autophagic activity. In vivo inhibition of this lncRNA led to an attenuated hypertrophic response and improved cardiac function through blockage of autophagy [84].

#### 3.2.2. ncRNAs in Cardiac-Calcium Handling

Alterations in genes encoding for calcium-handling proteins result in abnormal cardiac contractile function. Although intracellular levels of Ca^2+^ increase during hypertrophy, to facilitate contractile function, in physiological hypertrophic conditions there is an effective calcium handling by sarco/endoplasmic reticulum Ca^2+^-ATPase (SERCA2a), which is responsible for pumping Ca^2+^ back into the sarcoplasmic reticulum (SR) [1,85]. In dysfunctional hearts, however, this regulatory mechanism is impaired, due to abnormalities in SERCA2a levels [86]. Additionally, Ca^2+^ is necessary to initiate Ca^2+^-dependent signaling pathways, such as the calcineurin/NFAT signaling and the CaMKII signaling [17], and unbalanced calcium handling is a well-established driving force towards the development of cardiac dysfunction and heart disease.

miR-146a, miR-328, miR-25, and the lncRNA myocardial-infarction-associated transcript (MIAT) are known to disturb SERCA2a-dependent pathways. miR-146a causes abnormal Ca^2+^ handling in cardiomyocytes in pressure-overload mouse models, mainly through deregulation of small ubiquitin-like modifier 1 (SUMO1) and the consequent reduced SERCA2a SUMOylation, leading to impaired contractility [87]. Furthermore, miR-328, miR-25, and MIAT promote cardiac hypertrophy by downregulating SERCA2a, and thus, impairing calcium handling [88,89]. In vivo inhibition of these four ncRNAs resulted in attenuated hypertrophy in mice subjected to TAC, which may be, to some extent, linked to improved calcium handling and enhanced cardiomyocyte contractility [90].

A study by Xu and colleagues demonstrated that circHIPK3, a circular RNA produced by the third exon of the Homeodomain Interacting Protein Kinase 3 (HIPK3) gene, sponges miR-185-3p in cardiac hypertrophy. This results in upregulation of the calcium-sensing receptor (CaSR), with consequent impairment of calcium handling and severe cardiac remodeling. Silencing of this circRNA inhibited hypertrophic growth and cardiac dysfunction in mice [91].

#### 3.2.3. ncRNAs in Cardiac Metabolic Changes

Metabolic changes, inherent to pathological hypertrophy, consist of using glucose in place of fatty acid oxidation to produce ATP and satisfy the heart’s energetic demands [1,92]. In this way, the production of ATP requires less oxygen, which may be preferable in hypertrophic circumstances induced by workload increase [93]. Once the energy requirements exceed the energy supply, simultaneous expression of different metabolic gene programs fails to counterbalance, contractility becomes compromised, and the progression towards heart failure begins, with myocardial ATP levels reduced to more than half [94,95].

miR-27b seems to play a role in cardiac hypertrophy-associated metabolic changes. By targeting and downregulating fibroblast growth factor 1 (FGF1), a mitochondrial oxidative phosphorylation (OXPHOS) enhancer, miR-27b-3p aggravates pressure overload-induced cardiac hypertrophy in mice. Both the inhibition of this microRNA and the administration of recombinant FGF1 (rFGF1) resulted in improved mitochondrial OXPHOS and attenuated pathological cardiac remodeling [96].

On the other hand, miR-214 aggravates cardiac hypertrophy by targeting and inhibiting sirtuin 3 (SIRT3), impairing mitochondrial function and perturbing energy metabolism. While overexpression of miR-214 leads to mitochondrial damage and cardiac hypertrophy in mice, its silencing attenuates mitochondrial injury and decreases the hypertrophic response [97].

#### 3.2.4. ncrNAs in Cardiac Fibrosis

Fibroblasts, as an important cellular component of the cardiac tissue, are involved in maintaining homeostasis, both in physiological and stress conditions. Under chronic stress, a pro-inflammatory environment develops, and cardiac fibroblasts can transit to myofibroblasts, the predominant phenotype, upon cardiac injury and fibrosis. Myofibroblasts are, persistently, activated, and proliferate and regulate a positive feedback loop of transforming growth factor -β (TGF-β) secretion [98]. The result is an abnormal deposition and accumulation of collagen in the heart and, ultimately, mechanical stiffening, which is intimately related to cardiac dysfunction and arrhythmogenesis, by impaired conduction [1,99].

The involvement of miR-21 in cardiac disease has been broadly investigated, with several studies showing a clear discrepancy in expression profiles, from healthy and failing hearts. Different targets have been already described for this microRNA, highlighting its complexity. In 2008, miR-21 was found to increase ERK-MAP kinase activity in fibroblasts, impacting their structure and function, and controlling the extent of interstitial fibrosis. In a mouse model of pressure overload, cardiac function was improved, when this miRNA was silenced by a specific antagomiR [100]. Some years later, miR-21 was found to be upregulated by TGF-β, leading to silencing of PTEN and activation of the Akt signaling pathway. Again, treatment of mice hearts with a specific antagomiR attenuated fibrotic effects mediated by endothelial-to-mesenchymal transition (EndMT) [72]. Further studies showed that miR-21 passenger strand, miR-21-3p, was enriched, in cardiac-fibroblast-secreted exosomes. This strand is reported to target and inhibit sorbin and SH3 domain-containing protein 2 (SORBS2) and PDZ and LIM domain 5 (PDLIM5), inducing cardiomyocyte hypertrophy. Pharmacologically inhibition of miR-21-3p in mouse hypertrophied hearts, resulted in attenuated cardiac phenotypes, corroborating previous findings [101]. Recently, miR-21 was inhibited, with a specific antimiR in a pig model of ischemia/reperfusion injury, resulting in reduced cardiac fibrosis and hypertrophy, with an overall improvement of cardiac function, highlighting the translational potential of strategies targeting this microRNA [102].

miR-1 was shown to reverse pressure overload-induced cardiac hypertrophy in rats and attenuate pathological remodeling, by targeting Fibullin-2 (Fbln2), a protein involved in extracellular matrix remodeling. The cardioprotective effects of miR-1 result from a combination of events, including improvement of calcium handling, inactivation of MAPK, and improvement of extracellular matrix remodeling [103]. Similarly, the miR-221/222 family was described to play a cardiac protective role, by downregulating profibrotic signaling pathways through downregulation of JNK1, a member of the MAPK signaling pathway. JNK1 is a regulator of TGF-β signaling pathway and mediates the expression of profibrotic genes. In agreement, inhibition of miR-221 and miR-222 in a mouse model of pressure-overload-induced hypertrophy led to increased fibrosis, with worsened left ventricular dilation and dysfunction [104].

#### 3.2.5. ncRNAs in Cardiac Inflammation

An increase in inflammation is a hallmark of pathological cardiac hypertrophy, which may exacerbate the pathology, through mechanisms that are not, yet, fully understood. While the initial short-term inflammatory response is beneficial for cardiac repair [105], chronic inflammation is undesirable, as it results in tissue damage, defective cardiac remodeling, and cardiac dysfunction [106]. Such a pathological scenario is, often, a result of the induction and activation of different cytokines and chemokines including TNF-α, interleukin (IL)- 1 and 6, interferon (IFN)-γ, and the chemokine C-C motif ligand 2 (CCL2). These mediators promote progressive cardiac remodeling and dysfunction, by impairing cardiac contractile function, cardiomyocyte apoptosis, fibrosis, angiogenesis, and different signaling pathways involved in cardiac disease [107].

Interestingly, miR-27b negatively regulates the expression of peroxisome proliferator-activated receptor-γ (PPAR-γ), which was previously suggested as a cardiac protector, possibly by inhibiting the expression of TNF-α. This suggests that the deleterious role of miR-27b in cardiac hypertrophy can be related to an increase in inflammatory cytokines [108,109].

The lncRNA cardiac-hypertrophy-related factor (CHRF) was found upregulated in hypertrophic mouse hearts, as well as in samples from human HF patients. CHRF is described to downregulate the expression of miR-489 and, as result, upregulate the myeloid differentiation primary response gene 88 (Myd88), identified as an inflammatory mediator and a pro-hypertrophic factor, in mouse hearts. By sponging miR-489 and increasing the levels of Myd88, CHRF facilitates the onset of hypertrophy. In line, enforced expression of this lncRNA leads to pathological remodeling and increased cardiomyocyte death, while its knockdown, through adenoviral CHRF-siRNA injections, attenuated cardiac hypertrophy in mice [110].

Macrophages are inflammatory cell types, described to play a crucial role in cardiac remodeling. Dysregulation of their polarization between the pro-inflammatory (M1) and anti-inflammatory (M2) phenotypes is being extensively studied in heart disease [111]. miR-155 expression in macrophages is reported to promote cardiac inflammation and, consequently, hypertrophy and dysfunction, in response to pressure overload. Expression of this microRNA in macrophages is associated with pro-inflammatory M1-type stimuli, including TNF-α and IFN-γ, and it is thought to be involved in macrophage activation. miR-155 was found to target the suppressor of cytokine signaling 1 (SOCS1), promoting pro-inflammatory cytokine signaling, and its ablation in mice subjected to pressure overload resulted in decreased inflammation, hypertrophy, and cardiac dysfunction [112].

#### 3.2.6. ncRNAs in Cardiac Angiogenesis

Cardiac pathological remodeling is, often, associated with the myocardium’s inability to maintain efficient perfusion and nutrient supply, as it grows [1,17]. Capillary rarefaction occurs, only under pathological conditions, and interrupts the coordination between hypertrophic growth and angiogenesis, progressing towards irreparable function loss [113]. Enhancement of myocardial angiogenesis, under stress conditions, showed improved outcomes in the development of heart failure [114,115]. In this context, miR-195-3p seems to accelerate the transition from cardiac hypertrophy to heart failure in pressure overload conditions, by regulating AMPKα2/VEGF signaling, and, thus, impairing angiogenesis. Inhibition of this microRNAs in mice was achieved with specific antisense oligonucleotides, with consequent cardioprotective effects [116].

Table 3 recapitulates the ncRNAs that have been described to be specifically involved in the different pathophysiological mechanisms described.

## 4. Therapeutic Delivery of Drugs Targeting Non-Coding RNAs

Modulation of ncRNA expression has showed promising results in improving cardiac hypertrophy, in several models of cardiac disease. Table 1, Table 2 and Table 3 provide an overview of how therapeutic delivery of ncRNA stimulators or inhibitors are, currently, being developed and used in vivo.

### 4.1. Overexpression of ncRNAs

Therapies to overexpress ncRNAs in vivo, normally, consist of delivering miRNA mimics or viral vectors [24]. microRNA mimics are synthetic, double-stranded RNA fragments that mimic the mature sequence of the microRNA to overexpress. Similar to the endogenous microRNAs, mimics, also, result in a guide strand that will be functional. Although their therapeutic potential is being recognized, some challenges arise regarding their systemic activity, as once they reach off-target regions of the body, the overexpression of a microRNA in organs, where it is not endogenously expressed, may lead to adverse effects. Moreover, these molecules may induce an inflammatory response by stimulating the immune system [117]. To overcome these limitations, microRNA mimics can be chemically modified to be more specific, stable, and efficient. Linking these to RNA molecules able to specifically bind to target receptors, known as aptamers, is another strategy to increase cell-type-specific uptake [24,118].

The use of viral vectors is another common approach, to increase the expression of microRNAs as well as ncRNAs, in general. Lentiviruses or adenoviruses are typical choices, and they, normally, carry the microRNA precursor, instead of the mature microRNA sequence, leading to its overexpression. As this will result in the generation of two microRNAs and overexpression of both strands in an unrestrained fashion, the consequent response is difficult to predict [24]. On the other hand, AAV vectors constitute a widely explored platform for gene therapy delivery, and great results have been achieved in the delivery of RNA therapeutics. AAV vectors, containing capsid proteins of serotype 9 (AAV9), show improved cardiac specificity for gene delivery in rodents, while AAV6 seems to be the major cardiotropic for delivery in large animal models [119]. AAV9 vectors expressing cardioprotective microRNAs have been used in therapeutic approaches to increase expression of miR-106b~25 cluster [40], miR-1 [103], miR-378 [69], miR-148a [74], miR-29 [75], miR-217 [73], miR-146a [87], lncRNA Gm15834 [84], and circSlc8a1 [54]. However, limitations and worrying effects are emerging from the use of this approach, in preclinical research. AAV6-mediated delivery of miR-199a in infarcted pigs led to cardiomyocyte de-differentiation and proliferation, with consequent improvement of cardiac function. These beneficial results were haunted by the long-term, uncontrolled response to the overexpression of this miRNA, with most pigs dying of sudden death after eight weeks [120]. Moreover, human trials have shown disappointing efficiency for AAV-mediated gene therapy, as is the case of CUPID 2, a phase 2b trial in patients with HF and reduced ejection fraction, showing that, although without safety concerns, AAV1/SERCA2a therapy was unable to improve time to recurrent events, compared with a placebo [121].

### 4.2. Inhibition of ncRNAs

Therapeutic delivery of ncRNA inhibitors is, mainly, achieved by siRNAs and antisense oligonucleotides (ASOs).

siRNAs consist of a duplex of two 21-nucleotide RNA molecules, where 19 nucleotides are bases totally complementary to target transcripts, and the other two oligonucleotides are terminal 3′overhangs. To have an RNA interference function, siRNAs, similarly to microRNAs, need to be loaded into the RNA-interfering silencing complex (RISC). However, contrarily to microRNAs, after being perfectly processed, a siRNA can be loaded onto RISC without the mediation of Tat–RNA-binding protein (TRBP)/PACT (PKR activating protein)/Dicer complex [122]. The complete complementarity between siRNA and its target transcript mediates the degradation of the target mRNA, resulting in gene silencing [24,123]. Currently, there are several FDA-approved siRNAs, but none of these are aiming at cardiovascular disease [124,125,126]. The agent inclisiran, which is, so far, the closest to the cardiovascular field, is a siRNA drug for lowering LDL Cholesterol in patients with atherosclerotic cardiovascular disease (ASCVD) that was approved by the European Medicines Agency (EMA) in 2020 [127] but has not, yet, been approved by the US FDA [128]. siRNAs can also be expressed by viral vectors, which may improve oligonucleotide delivery. Moreover, small hairpin RNAs (shRNAs) can, also, be used to mediate the silencing of ncRNAs, as improved efficacy was reported. shRNAs are RNA fragments, usually about 80 nucleotides in length, synthesized in the nucleus of cells, which will be processed to form siRNAs. For this processing, shRNAs assimilate into the endogenous miRNA pathway, with the help of the TRBP/PACT/Dicer complex, to ensure an appropriate size and form. This mediation is believed to result in a more efficient loading process onto RISC, compared to siRNA’s loading process [122]. shRNAs can be continuously synthesized by the host cells, with a more prolonged effect compared to siRNAs, often degraded after 48 h [122,129]. Effective knockdown is, also, achieved with lower concentrations of shRNAs, which makes them less prone to off-target effects [122]. Viral vector delivery of these shRNAs resulted in better integration and, consequently, prolonged knockdown of the target ncRNAs [130]. Although siRNAs and shRNAs are both promising tools, both have common drawbacks. If, on one hand, the effect of shRNA is more durable, siRNAs’s transient nature may be preferrable in some situations, with safety being a balance between dosage and period of delivery. Moreover, the endogenous processing machinery of shRNAs is more difficult to chemically modify and improve than chemically synthesized siRNA, whose efficacy can be optimized [122].

ASOs have, also, been extensively investigated for their therapeutic potential in silencing ncRNAs. These are synthetic, small (between 18 and 30 nucleotides), single-stranded oligonucleotides, whose mechanism of action may vary and, according to how they mediate gene silencing, can be subdivided in RNase-H-competent ASOs and steric-block ASOs [123]. RNase-H-competent ASOs bind to a specific mRNA target, forming a duplex that will be recognized by RNAse H and cleaved, which leads to degradation of the target transcript and, consequently, its silencing. This subtype of ASO, usually, follows a gapmer pattern: their structure consists of a central DNA sequence (“gap”), long enough to induce RNase H-mediated cleavage, complemented with RNA-based flanking regions in both ends, promoting the binding to the target region [123]. “Gapmer” is the most widely used term to refer to chimeric RNase-H-competent ASOs and, contrarily to other approaches, such as siRNAs and other ASOs, they can enter the nucleus and mediate target mRNA degradation there, with RNase H being active both in the cytoplasm and in the nucleus. Thus, gapmers are, generally, used to target and inhibit lncRNAs, which are preferentially located in the nucleus [24,123]. Some RNase-H-competent ASOs are already approved [131], but, again, they target other conditions than cardiovascular diseases. However, there are still some challenges inherent to this approach, since hepatoxicity was reported after RNase-H-mediated mRNA degradation in off-target transcripts [123]. Steric-block ASOs, in turn, block the access to pre-mRNA and mRNA specific transcripts but do not induce their degradation by RNase H [123]. By perfect complementarity, these ASOs bind to their specific miRNA sequences, with high affinity, inhibiting further RNA-RNA and/or protein-RNA interactions in this region. These are, commonly, termed anti-miRNA oligonucleotides (AMOs) or anti-miRs, and they have been broadly explored [24,123]. An AMO targeting an ncRNA in cardiovascular disease is, currently, in a clinical phase; CDR132L, developed by Cardior Pharmaceuticals GmbH, is an antimiR able to inhibit the cardiac deleterious miR-132. Phase 1 of NCT04045405 in patients with HF of ischemic origin has shown very promising results [132].

Different types of AMOs have been defined, according to the chemical modifications of their original structure. AntagomiRs are a very popular type of AMO that have been, extensively, used in preclinical research targeting ncRNAs (Table 1, Table 2 and Table 3). These AMOs are modified to include 2′-O-(metoxyethyl) (2′MOE) nucleic acids, linked with phosphorothioate (PS) bonds at both ends of the molecule, and a cholesterol group attached to the 3′ end. Such modifications will be further described below, but they mainly aim at increasing oligonucleotide stability in circulation, conferring protection from exonuclease activity, and facilitating delivery [133].

### 4.3. Strategies to Improve Oligonucleotide Drug Delivery

Despite their therapeutic potential, with an increasing number of promising preclinical results, oligonucleotide drug delivery is still facing some limitations, either when the goal is to overexpress or silence an ncRNA. One of the most troublesome aspects is the need for high doses to achieve a therapeutically relevant result, which, often, leads to adverse effects and toxicity, narrowing further development towards clinical trials [24]. Moreover, multiple dosing is, often, necessary to achieve and maintain a sustainable result, which may be difficult to coordinate in approaches where the physician needs to play a role. To increase the effectiveness of these oligonucleotides, as well as to prolong their action and avoid multiple administrations, further improvement of their stability and resistance to degradation is, still, needed. Finally, the specificity of the oligonucleotides is a very important factor, since their accumulation in the kidney and other off-target organs has been reported [134]. The multitarget nature of ncRNAs and their ability to regulate different signaling pathways, usually involved in crucial processes, means that a change in their expression in unpredictable regions may have devastating effects, which makes unspecific delivery a major concern [24,135]. In a concrete example, regulating the expression of ncRNAs to increase cell proliferation in the heart, when reaching off-target organs, may result in dysregulated cell proliferation and consequent tumor growth [24]. Chemical modifications of the oligonucleotides and their encapsulation in lipid or nanocarriers are current strategies to overcome certain of the identified limitations, which are still haunting oligonucleotide drug delivery.

#### 4.3.1. Chemical Modifications

Specific chemical modification of oligonucleotides has been described to improve their pharmacokinetics, pharmacodynamics, and biodistribution [123]. Currently, the most explored strategies are backbone modifications, ribose sugar modifications, bridging of the nucleic acids, and bioconjugation.

Backbone modifications, mostly, include the incorporation of phosphorothioate groups (PS), where a non-bridging oxygen from the phosphate backbone is replaced with a sulfur atom. Oligonucleotides, containing PS linkages, present a greater resistance to nucleases, as well as a greater ability to bind to plasma and intracellular proteins, promoting their accumulation and increasing their circulation time [123,133]. Despite the improved life span of the oligonucleotide, this modification can reduce binding affinity for its target, leading to the preferrable combination of different modifications [123].

Ribose sugar modifications are, also, very commonly applied. These modifications, usually, occur at the 2′ position of the ribose sugar, and they, often, consist of 2′-O-methyl (2′-OMe), 2′-O-methoxyethyl (2′-MOE), and 2′-Fluoro (2′-F) alternates. By exchanging the 2′-hydroxyl group of the RNA ribose, the oligonucleotide benefits from increased stability in plasma, longer tissue life span, and higher binding affinity [123,133]. Additionally, 2′-OMe modifications at key positions can prevent immunogenic effects associated with the administration of ASOs and siRNAs, since they can “camouflage” their identity and interfere with their recognition by Toll-like receptors [136,137]. 2′-OMe modifications have been broadly applied to test ncRNA modulation in cardiac hypertrophy (Table 1). AntagomiRs carry this modification, and their therapeutic potential has been highlighted, being one of the most relevant approaches to inhibit the expression of microRNAs in vivo. Potent inhibition of miR-122 with AMOs containing 2′-OMe [138], 2′-MOE [139] and 2′-F [140] modifications was successfully achieved, but the use of additional modifications, such as the use of bridged nucleic acids, seem to increase their advantage [123,135].

Bridging of nucleic acids consists of connecting 2′-oxygen to the 4′-carbon of the ribose sugar, with a methylene bridge, forming a 3′-endo conformation (bicyclic sugar moiety). Locked nucleic acid (LNA) is the most common type of bridged nucleic acid (BNA), and this approach has been demonstrated to enhance oligonucleotide nuclease stability and the affinity for their target [133,135]. Moreover, LNA oligonucleotides are highly soluble and display low toxicity at low doses [123]. Some adverse effects were, however, described for LNAs, including in vivo hepatoxicity [141] and off-target microRNA inhibition [142]. Nevertheless, LNAs are a promising strategy, and they seem to be well tolerated in vivo [135].

Bioconjugates are single-component, homogeneous molecular entities, easy to synthesize, and with well-defined pharmacokinetic properties. They are typically small, which favors biodistribution profiles and allows them to reach less accessible tissues, such as the nervous system and lungs, with continuous nonfenestrated endothelium [123]. Bioconjugation, usually, aims to promote the interaction between the bioconjugate and its target cell surface receptor protein, facilitating internalization and enabling targeted delivery [123]. Cholesterol conjugates are common: they are a specific type of lipid conjugates, where a cholesterol group is added to one of the ends of the oligonucleotide, facilitating the interactions between the oligonucleotide and the circulatory lipoprotein particles and promoting delivery [123,133].

The described chemical modifications are the most widely used to improve pharmacokinetic properties and specific delivery of oligonucleotides in a “naked” state, i.e., lacking additional delivery vehicles. Some other strategies to improve their properties include their encapsulation in microcarriers and nanocarriers, as well as the use of viral vectors. Furthermore, efficient and sustained delivery of oligonucleotides may benefit from device assistance.

#### 4.3.2. Encapsulation and Other Approaches

Encapsulation of oligonucleotide-based drugs, to improve their delivery, has received considerable attention and promising results are, already, described in vivo. Nanocarriers can be modified at both the biophysical and biological levels, not only allowing a full adjustment of their size, shape, physical and chemical composition but also for ligand functionalization, to improve targeting [143]. Lipid-based nanocarriers are being commonly used to encapsulate antisense drugs and improve their pharmacokinetic properties. However, a drawback of using LNPs is their limitation to, mostly, deliver drugs in the liver and the reticuloendothelial system, due to their relatively large size [143,144]. The delivery potential of natural biological nanoparticles is, also, being explored. Exosomes are a class of extracellular vesicles that are thought to be released by all types of cells into the extracellular space, allowing intercellular communication, by transferring their contents among cells [145]. Several beneficial aspects of the use of exosomes in drug delivery are identified, including their resistance to phagocytosis [146], safety, and lack of toxicity [147] as well as their potential to be produced in an autologous way. The surface of engineered exosomes can, also, be modified to increase targeted delivery. A less common nanocarrier consists of spherical nucleic acids (SNA), to which surface, contrarily to other nanoparticles’s designs, drugs are attached, outwards from the core structure. Still, drugs are protected from degradation, due to steric impediment, protein interactions, and high local salt concentration [148].

In addition to nanoparticles, microparticles have been, also, described as useful platforms for antisense drug delivery. In the context of cardiac disease, intracoronary administration of miR-92a encapsulated in microspheres showed targeted delivery in the vasculature and consequent improvement of cardiac function in pigs [149]. Encapsulation is attracting much interest in the field of drug delivery, and the versatility and potential of both nanocarriers and microcarriers make them strong candidates to solve current issues, regarding oligonucleotide delivery.

Finally, device-based approaches can, also, help to improve the delivery of antisense drugs, and an initialstrategy was employed, to augment the drug’s concentration in the heart or vasculature. In a pig model of ischemia/reperfusion injury, catheter-based delivery of an LNA for the inhibition of miR-92a resulted in significantly improved cardiac function, compared to intravenous infusion. This approach allowed the use of a relatively low LNA dose [142], so, although the efficiency of delivery was high, further strategies to increase specificity and targeted delivery should be developed.

**Figure 2:**
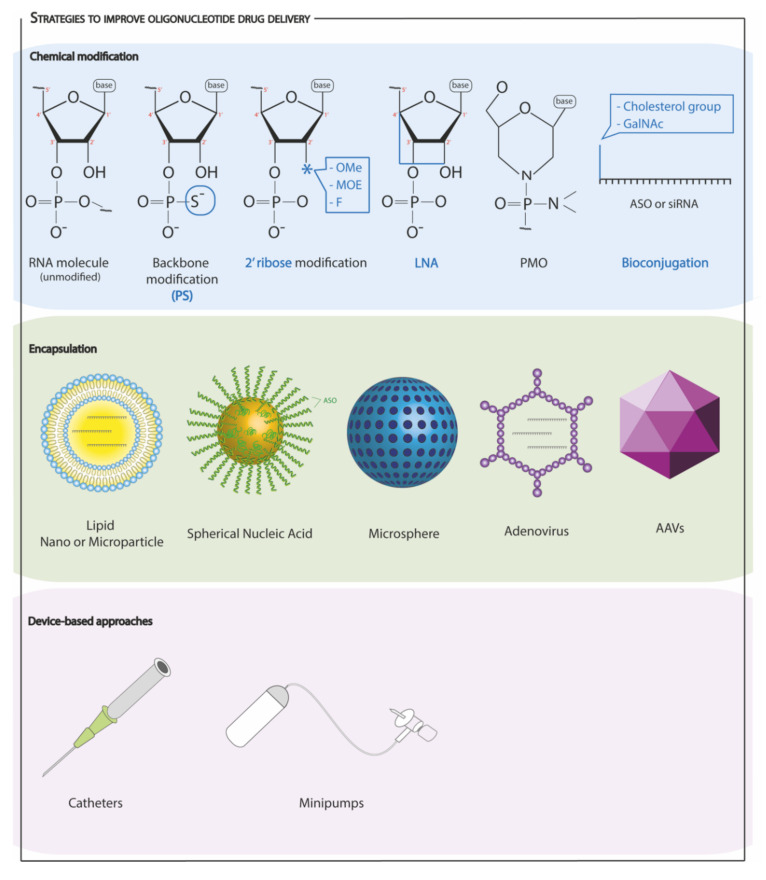
Different strategies to improve the delivery of oligonucleotide-based therapeutics.

Figure 2 schematically summarizes the above-mentioned strategies to improve oligonucleotide drug delivery.

## 5. Concluding Remarks

The number of approved nucleic acid-based therapeutics is rapidly increasing, which was, also, fueled by the COVID-19 pandemic, with efficient RNA-based vaccines being developed [150]. This may increase the credibility of such therapeutic approaches.

Although oligonucleotide drugs have already been approved, targeting some pathologies, there are still no approved drugs for the treatment of cardiovascular diseases, with heart delivery showing some challenges. However, clinical trials with antisense drugs for the treatment of HF are, already, being reported: CDR132L, developed by Cardior Pharmaceuticals GmbH, described earlier in this paper, as well as IONIS-AGT-LRx. IONIS-AGT-LRx, is currently under Phase 2 of clinical research (NCT04836182) for the treatment of chronic HF with reduced ejection fraction. This ASO, developed by Ionis Pharmaceuticals, Inc., is designed to target angiotensinogen (AGT) and, consequently, inhibit RAAS pathways, acting as a therapeutic approach for the treatment of hypertension and HF conditions. Promising results were described for the past clinical trials with this ASO, which include the assessment of safety, tolerability, and efficacy in hypertensive patients (phase 2, trials NCT04083222 and NCT03714776) and healthy volunteers (phase 1, trial NCT03101878) [151]. Although the latter ASO is not targeting ncRNAs, clinical research in antisense drugs for the treatment of cardiac diseases is, already, a reality. These trials will, hopefully, lead the way for many others targeting ncRNAs involved in cardiac hypertrophy.

A great number of these drugs are, currently, being preclinically studied in large animal models. However, and, although, the results seem promising, a major concern relies on the translation into clinical research, since the number of drugs failing clinical trials, due to poor translation, is disappointingly high. Thus, humanized models that better predict clinical outcomes are being established and are of major importance. Human ex vivo models include engineered heart tissues (EHTs) [152] and myocardial slice models [153] derived from human cells or tissues, which provide helpful approaches to predict human response as well asto reduce the number of animals in preclinical research [154]. Despite the great advantages of these approaches, humanized models accurately mimicking cardiac hypertrophy conditions are still lacking, since such pathological conditions seems challenging to obtain, in ex vivo conditions.

Another very important limitation of ncRNA-targeting therapies consists of the pleiotropy of ncRNAs: since they can target different signaling pathways, at the same time, in different organs, their modulation requires special attention to adverse off-target effects. Preclinical toxicity studies should play a critical role in the success of such therapies. These should, also, consider sex-related differences in therapeutic responses, as a sex-specific regulation of cardiac microRNAs has shown crucial importance in the context of cardiac hypertrophy [77,155].

Great challenges require great solutions and, in an era of fast advances for clinical research, it is expected that antisense drugs targeting ncRNAs in cardiac hypertrophy will, soon, find their place in clinical practice.

## Figures and Tables

**Table 1:** ncRNAs modulating gene expression programs involved in concentric cardiac hypertrophy.

ncRNA	Mechanism of Action	Modulation Strategy (In Vivo)	Ref
miR-92b-3p	↓ *MEF2D*	agomiR-92b-3p (↑)	[38]
miR-106~25 cluster	↓ MEF2D + Hand2	AAV9-miR-106b~25 (↑) AntagomiR- (-106b, -93, -25) (↓)	[40]
miR-145-5p	↓ ATF3 (↑ pro-hyp genes)	ad-circNfix (↑)	[41]
circNfix	Sponge for miR-145-5p (↑ ATF3; ↓ pro-hyp genes)		
lncRNA Mhrt	↓ Chromatin Remodeling; (↓ pro-hyp genes)	Mhrt779 transgenic mice (↑)	[43]
lncRNA Chaer	↓ *EZH2*; ↓ H3K27me (↑ pro-hyp genes)	siChaer (↓) Chaer KO mice (↓)	[44]

**Table 2:** ncRNAs involved in specific signaling pathways involved in cardiac hypertrophy.

ncRNA	Mechanism of Action	In Vivo Delivery or Gain/Loss of Function Strategy	Ref
**Calcineurin/NFAT Signaling**			
miR-1	↓ Cn/NFAT SP ^1^	Ad-miR-1 (↑)	[50]
miR-133		antagomiR-133 (↓) Ad133 (↑)	[52]
circSLC8A1-1	Sponge for miR-133 (↑ Cn/NFAT SP)	AAV9-circSlc8a1 (↑) AAV9-sh-circSlc8a1 (↓)	[54]
miR-182	↑ Cn/NFAT SP	miR-182 agomir (↑) CHO-PGEA/miR-182 (↑) CHO-PGEA/miR-182-in (↓)	[58] [59]
miR-23a		antagomiR-23a (↓)	[60]
miR-212/132		antagomiR-132 (↓)	[61]
miR-132		antimiR-132 (↓)	[62]
miR-199b		antagomiR-199b (↓)	[56]
piRNA CHAPIR	↓ m6A methylation of *Parp10* (↑ Cn/NFAT SP)	CHAPIR mimic (↑) CHAPIR antagomiR (↓)	[34]
**CaMKII Signaling**			
miR-675	↓ CamKII	antagomiR-675 (↓)	[65]
lncRNA H19	Sponge for miR-675 (↑ CamKII)		
lncRNA TINCR	↑ EZH2; ↑ H3K27me3 (↓ CamKII)	Lentivirus pcDNA-TINCR (↑)	[66]
miR-214	↓ EZH2	antagomiR-214 (↓)	[67]
**MAPK Signaling**			
miR-378	↓ MAPK SP	AgomiR-378 (↑) AntagomiR (↓) AAV9–miR-378 (↑)	[68,69]
miR-499	↑ MAPK SP	miR-499 transgenic mice (↑)	[70]
**Akt Signaling**			
lncRNA CHAR	Sponge for miR-20b	Lenti-CHAR (↑) Lenti-sh-CHAR (↓)	[71]
miR-20b	↓ PTEN; ↑ Akt SP		
miR-21		antagomiR-21 (↓)	[72]
miR-217		rAAV9-miR-217 (↑) rAAV9-miR-217-TUD (↓)	[73]
**JAK/STAT Signaling**			
miR-148a	↓JAK/STAT SP	AAV9-148a (↑) AntagomiR-148a-3p (↓)	[74]
**Wnt Signaling**			
miR-29	↑ Wnt SP	AAV9-miR-29a (↑) antimiR-29 (↓)	[75,77]

^1^ SP: Signaling Pathway.

**Table 3:** Overview of the ncRNAs reported to be specifically involved in the pathophysiological mechanisms inherent to cardiac hypertrophy.

ncRNA	Mechanism of Action	In Vivo Delivery or Gain/Loss of Function Strategy	Ref
**Cardiomyocyte remodeling and loss**			
mir-183-5p	↓ Apoptosis	miR-183 mimics (↑) ASO inhibitor (↓)	[78]
miR-223	↑ Apoptosis	Ad-HRCR (↑)	[79]
HRCR	Sponge for miR-223; ↓ Apoptosis		
lncRNA Chast	Regulation of the expression of adjacent protein (Plekhm1) (↓ Autophagy)	AAV9-Chast (↑) LNA GapmeR-Chast (↓)	[82]
miR-30b-3p	↓ Autophagy	AAV9-sh-Gm15834 (↓)	[84]
Gm15834	Sponge for miR-30b-3p (↑ Autophagy)		
**Calcium Handling**			
miR-146a	↓ SERCA2a SUMOylation; (↓ calcium handling)	rAAV9_premir-146a (↑) rAAV9_decoy-146a (↓)	[87]
miR-328	↓ SERCA2a (↓ calcium handling)	LNA-antimiR-328 (↓)	[88]
miR-25		AAV9-miR-25 (↑) Anti-miR-25 (↓)	[89]
lncRNA MIAT		MIAT KO mice	[90]
miR-185-3p	↓ CaSR; (↑ calcium handling)	ad-si-circHIPK3 (↓)	[91]
circHIPK3	Sponge for miR-185-3p; ↑ CaSR (↓ calcium handling)		
**Metabolism**			
miR-27b-3p	↓ FGF1; (↓ mitochondrial metabolism)	miR-27b-3p KO mice (↓)	[96]
miR-214	↓ SIRT3; (↓ mitochondrial metabolism)	agomiR-214 (↑) antagomiR-214 (↓)	[97]
**Fibrosis**			
miR-21	↑ Akt SP (↑ fibrosis)	antagomiR-21 (↓)	[72]
	↑ MAPK SP (↑ fibrosis)		[100]
	↓ SORBS2 ↓ PDLIM5		[101]
miR-1	↓ fibrosis	AAV9-miR-1 (↑)	[103]
miR-221/222	↓ fibrosis	AntagomiRs (↓)	[104]
**Inflammation**			
miR-27b	↑ TNF-α (↑ Inflammation)	antagomiR-27b (↓)	[109]
miR-489	↓ *Myd88* (↓ Inflammation)	miR-489-3p mimic (↑) antagomiR-489 (↓)	[110]
lncRNA CHRF	Sponge for miR-489 (↑ Inflammation)	Adenoviral CHRF (↑) Adenoviral CHRF siRNA (↓)	
miR-155	↓ SOCS1 (↑ Inflammation)	miR-155 KO mice (↓)	[112]
**Angiogenesis**			
miR-195-3p	↓ angiogenesis	miR-195a-3p mimic (↑) miR-195a-3p antagomiR (↓)	[116]

## Data Availability

Not applicable.
